# Diagnosis of pregnancy after conservative management for adnexal torsion due to ovarian hyperstimulation in the same cycle

**DOI:** 10.5935/1518-0557.20140014

**Published:** 2014

**Authors:** Barış Büke, Gülnaz Şahin, Sibel Demir, Ayşin Akdoğan, Erol Tavmergen, Ege T. Göker

**Affiliations:** 1 Department of Obstetrics and Gynecology, Ege University Faculty of Medicine, Izmir, Turkey; 2 Department of Infertility and IVF, Ege University Faculty of Medicine, Izmir, Turkey; 3 Department of Obstetrics and Gynecology, Alibey Hospital, Istanbul, Turkey

**Keywords:** Metabolome, embryo selection, embryo transfer

## Abstract

We report a case of right adnexal torsion during the embryo implantation period of an IVF/ICSI cycle. A 26-yearold woman who diagnosed as primary infertility was included in an IVF/ICSI program. In the following period right adnexal torsion occurred at the sixth day of the embryo transfer. Laparoscopic detortion was successfully performed with preserving the adnexia. The patient had positive pregnancy test in the subsequent week.

## INTRODUCTION

Ovarian hyperstimulation is a complication of ovulation induction with gonadotropins (Speroff & Fritz, 2005). Because of this, after two decades of complex fertility treatment regimens with high doses of hormones, simpler and more physiological protocols have been implemented in several IVF centers (Siristatidis *et al.*, 2009).

Adnexal torsion is an emergency condition where the adnex rotate on their pedicle compromising their blood supply. It is a rare cause of acute abdominal pain and a true gynecologic emergency (Hibbard, 1985). It accounts for approximately 3% of gynecological emergencies, and 10- 20% of ovarian torsions ocur during pregnancy, frequently occurring in the first trimester after ovarian stimulation for IVF (Roest *et al.*,1996; Govaerts *et al.* 1998; Gorkemli *et al.*, 2002).

Here, we report a case of unilateral adnexal torsion before the diagnosis of pregnancy after an ICSI embryo transfer.

## CASE PRESENTATION

A 26-year-old healthy woman with a history of primary infertility for 4 years and polycystic ovarian disease was referred to our Center of Reproductive Medicine and Assisted Reproduction in March 2011. The patient had the typical clinical and echographic criteria of polycystic ovarian syndrome (PCOS) (The Rotterdam ESHRE/ASRM-Sponsored PCOS Consensus Workshop Group, 2004). Furthermore, in 2011, a diagnostic laparoscopy revealed patent tubes and PCO. The male partner had normal semen parameters according to World Health Organization (WHO) standards (Cha *et al.*,2005).

In June 2011, at our center, the couple was included in an intrauterin insemination (IUI) program. The patient underwent ovulation induction with 75 IU/day FSH, but she could not achieved pregnancy. In November 2011, with 75 IU/day FSH, a new IUI program was tried but she had multipl follicules so the cycle was cancelled.

In January 2012, at our center, the couple was included in an In Vitro Fertilization (IVF) program. Controlled ovarian hyperstimulation (COH) was performed using a flexible GnRH antagonist protocol with mild stimulation that involved the administration of recombinant FSH (rFSH) (Gonal-F; Serono, Geneva, Switzerland), 150 UI daily, from day three of spontaneous menstrual cycle. During ultrasound monitoring, when at least one follicle reached 14 mm in diameter, GnRH antagonist (Orgalutran 0.25 mg; Organon, Italia) 0.25 mg/day was added subcutaneously. The peak E2 value was 1938 pg/ml and the peak LH was: 2.8 pg/ml. In that cycle 21 oocytes were collected, ICSI method was used for insemination for 20 MII oocyte. Single embryo with good quality was transferred on day 3 after oocyte retrieval.

**Figure 1:**
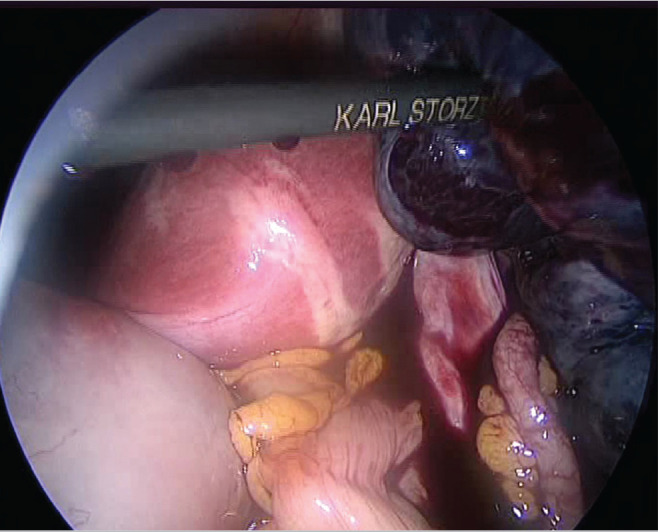
Laparoscopic photograph of right adnexal torsion at the utero-ovarian pedicle.

**Figure 2:**
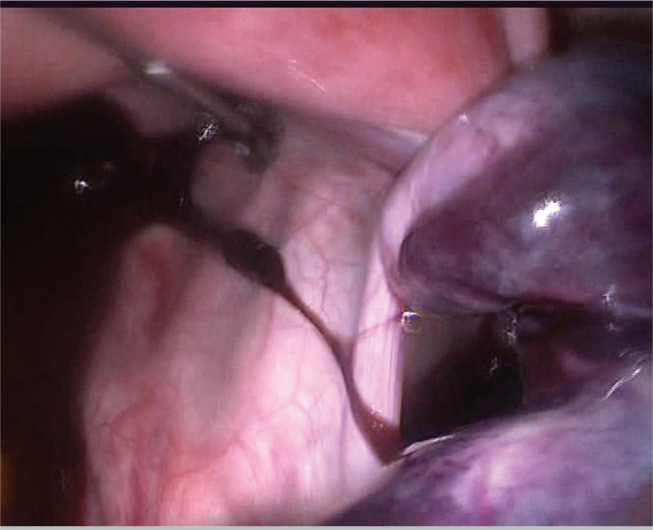
Underwind the twisted adnex, by pushing the ovary in the opposite direction of the torsion.

**Figure 3:**
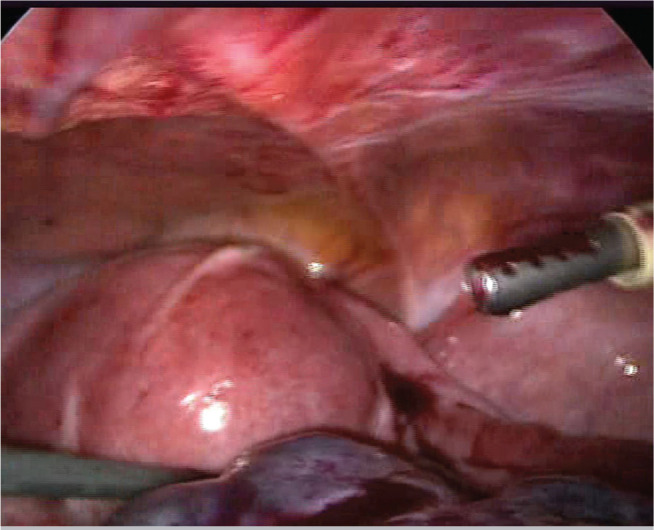
Recoloration and a decrease of the adnexal edema, suggesting a successful recovery.

Five days after embryo transfer, the patient applied to our clinic complaining of lower abdominal pain. She was afebrile and physical examination showed a point of maximal tenderness in the right lower abdominal quadrant. There was no vaginal bleeding nor any bowel symptoms. Acute appendicitis and renal colic were excluded. The laboratory workup showed a white blood cell count of 14.260/mm^3^ whereas hepatic enzymes, hematocrit and urine analysis were normal. Transvaginal ultrasound was carried out showing enlarged (10 × 7.5 cm) right ovary within coexistent mass, a small amount of fluid was revealed in the pouch of Douglas. The Colour Doppler examination was normal with presence of ovarian vascular flow bilaterally. Six hours after admission to the hospital, a worsening of clinical symptoms showed an acute abdominal pain with an abdominal mass reaching up to one cm away from umbilicus. In view of these findings a diagnostic laparoscopy was carried out. It was highly difficult to enter the abdomen. The laparoscopic findings showed a two times twisted right adnex with an ischemic ovary (Figure 1). The enlarged cystic ovary had a bluish hue and measured about 14 cm in diameter and the left ovary was about 7-8 cm in diameter and hyperstimulated. We proceeded to underwind the twisted adnex, by pushing the ovary in the opposite direction of the torsion (Figure 2). Aproximately, after 15 minutes, there was recoloration and a decrease of the adnexal edema, suggesting a successful recovery (Figure 3), and the ovary was preserved. The patient had an unremarkable postoperative course and was discharged in the following day.

After 12^th^ day of ET the beta HCG level was 58mIU/ml, progesterone level was 20.7mIU/ml and E2 level was 295mIU/ ml. Two weeks later transvaginal ultrasound revealed a live intrauterine pregnancy of six weeks gestational age. Follow up was driven at the perinatology department uneventfully with normal screening tests and fetal growth. The placenta was lying into the low uterine segment without reaching to internal servical os. But at the 38th week of pregnancy, she applied to our clinic with the complaint of vaginal bleeding. A bradicardic fetal heart rate was detected and immediate C/S section was performed. The fetus was delivered with no vital signs. There was no abnormality except the lower uterine segment site placenta at the operation.

## DISCUSSION

Polycystic ovary syndrome (PCOS) is the most common endocrine abnormality in reproductive-aged women and accounts for most of the anovulatory infertility (Speroff & Fritz, 2005). PCOS is often associated with infertility as a result of ovulatory irregularity. Some of these patients will require in vitro fertilization (IVF) to manage their infertility. Ovarian stimulation of the patient with PCOS for IVF might be challenging. Most patients have a robust response to gonadotropin stimulation and are at significant risk for hyperstimulation. Recent studies show that in vitro maturation (IVM) can be a potentially useful method for women with PCOS-related infertiliy. However, there are no adequately controlled studies available (Hibbard, 1985; Roest *et al.*, 1996; Govaerts *et al.*, 1998; Gorkemli *et al.*, 2002; The Rotterdam ESHRE/ASRM-Sponsored PCOS Consensus Workshop Group, 2004; Cha *et al.*, 2005; Siristatidis *et al.*, 2009).

Adnexal torsion is an emergency condition where the adnex rotate on their pedicle. Torsion of the right adnex is more common, and few reports of bilateral torsion, either simultaneously or subsequently, exist (Peña *et al.*, 2000; Mathevet *et al.*, 2003; Born *et al.*, 2004). Adnexal torsion frequently occur in the first trimester after ovarian stimulation for IVF (Hibbard, 1985; Roest *et al.*, 1996; Govaerts *et al.*, 1998; Gorkemli *et al.*, 2002). The diagnosis of ovarian torsion is difficult and occasionally remains a diagnostic dilemma. The clinical symptoms could be confused with other acute abdominal conditions. The signs of adnexal torsion include a palpable pelvic mass, signs of localized peritoneal irritation, a low grade fever, and leukocytosis. Preoperative diagnosis is difficult, especially in pregnant women. When complete torsion with hemorrhagic necrosis is suspected, immediate surgery is necessary. Ultrasonography is the primary imaging modality for evaluation of ovarian torsion.

Ultrasonography features of ovarian torsion include a unilateral enlarged ovary, uniform peripheral cystic structures, a coexistent mass within the affected ovary, free pelvic fluid, lack of arterial or venous flow, and a twisted vascular pedicle. The presence of flow at colour doppler imaging does not allow exclusion of torsion but suggests that the ovary may be viable, especially if flow is present centrally.

Absence of flow in twisted vascular pedicle may indicate that the ovary is not viable (Born *et al.*, 2004). Doppler sonography, although highly specific, has low sensitivity, as it may miss the diagnosis in approximately 60% of cases (Peña *et al.*, 2000; Born *et al.*, 2004).

Adnexal torsion is a surgical emergency during pregnancy. In the past, the traditional treatment was adnexal removal. Nowadays cases of adnexal torsion occurring during the first trimester of pregnancy should preferably undergo laparoscopy, which is suitable for diagnosis, evaluation and treatment (Mathevet *et al.*, 2003; Lo *et al.*, 2008). The main specific complications of laparoscopy during pregnancy are related to possible injury to the enlarged uterus and ovaries situated outside the pelvis and to the cardiovascular and respiratory alterations introduced by the pneumoperitoneum pressure and CO2 absorption. After laparoscopic detortion, 24 hours of postoperative observation is recommended.

As a conclusion, ovarian torsion is one of the complications of IVF/ICSI treatments and it should be kept in the mind that in case of adnexal torsion even after embryo transfer, laporoscopic management can be the appropriate approach.
